# Transverse Crack Detection in 3D Angle Interlock Glass Fibre Composites Using Acoustic Emission

**DOI:** 10.3390/ma9080699

**Published:** 2016-08-16

**Authors:** Matthieu Gresil, Mohamed Nasr Saleh, Constantinos Soutis

**Affiliations:** 1 i-Composites Lab, School of Materials, University of Manchester, 79 Sackville Street, Manchester M1 3NJ, UK; 2 National Composites Certification and Evaluation Facility, University of Manchester, Manchester M1 3NJ, UK; mohamed.saleh-7@postgrad.manchester.ac.uk; 3 Aerospace Research Institute, University of Manchester, Manchester M1 3NJ, UK; constantinos.soutis@manchester.ac.uk

**Keywords:** acoustic emission, Lamb waves, multi-physics finite element, piezoelectric sensors, 3D woven composite materials, structural health monitoring, transverse cracking

## Abstract

In addition to manufacturing cost and production rates, damage resistance has become a major issue for the composites industry. Three-dimensional (3D) woven composites have superior through-thickness properties compared to two-dimensional (2D) laminates, for example, improved impact damage resistance, high interlaminar fracture toughness and reduced notch sensitivity. The performance of 3D woven preforms is dependent on the fabric architecture, which is determined by the binding pattern. For this study, angle interlock (AI) structures with through-thickness binding were manufactured. The AI cracking simulation shows that the transverse component is the one that leads to transverse matrix cracking in the weft yarn under tensile loading. Monitoring of acoustic emission (AE) during mechanical loading is an effective tool in the study of damage processes in glass fiber-reinforced composites. Tests were performed with piezoelectric sensors bonded on a tensile specimen acting as passive receivers of AE signals. An experimental data has been generated which was useful to validate the multi-physics finite element method (MP-FEM), providing insight into the damage behaviour of novel 3D AI glass fibre composites. MP-FEM and experimental data showed that transverse crack generated a predominant flexural mode A0 and also a less energetic extensional mode S0.

## 1. Introduction

Fibre-reinforced composite materials are used extensively in the aerospace industry because of their light weight, superior corrosion resistance and improved fatigue properties when compared to metals. However, the manufacturing costs, production rates and damage resistance are current challenges faced by the composite industry. Three-dimensional (3D) woven composites have better through-the-thickness properties in comparison to two-dimensional (2D) laminates; they show damage resistance, high inter-laminar fracture toughness and reduced notch sensitivity that demonstrate a better damage tolerance. 3D fabrics were introduced to produce structural composites capable of withstanding multidirectional stresses. 

Monitoring of acoustic emission (AE) during mechanical loading is an effective and widely used tool in the study of damage processes in glass fiber-reinforced composites. This study provides further insight into the AE monitoring of 3D AI glass fibre composites. Tests were performed with piezoelectric sensors bonded on a tensile specimen acting as passive receivers of AE signals. These signals are carefully analysed to identify resin cracks in the warp yarn and relate to crack density.

### 1.1. Damaged Monitored by Acoustic Emission in Composite Materials

AE is a passive SHM technique that can be used for many applications. When crack grows, energy is released at the crack tip in form of waves. AE sensors can be used to measure these waves. Several sensors in combination can be used to estimate the severity of the crack and its location. Most publications show results from fatigue cracks in bulk materials and qualitative results from real structures. However, there is limited literature presenting quantitative results from plate-like structures and a lot of the experiments are based on simulated AE sources, e.g., pencil lead breaks [1]. One aim of this paper is to analyse the elastic waves generated from transverse cracks (TC) in a 3D angle interlock composite structures subjected to tensile loading. FEM can be used to model the AE waves from transverse crack and it can provide a better understanding of the AE generated from TC in composite plates.

The AE method allows the detection and location of damage using specific localisation algorithms. Knowledge of the propagation velocity and attenuation of the AE wave is required. However, contrary to metallic material, the anisotropic nature of composite material gives a large range of propagation velocity due to fibre orientation. Moreover, the attenuation of the AE waves is more complex than in a homogeneous material [2]. In addition, in a same composite material, wave attenuation is more significant in cracked than in healthy state, which will complicate the signal processing after few damage modes have developed, especially for the amplitude distribution. Qualifying damage started first in 2D composites and Mehan and Mullin in 1968 [3] managed to identify three basic failure mechanisms: (i) fiber fracture; (ii) matrix cracking; (iii) and fibre/matrix interfacial debonding. The authors reported the application of AE in composites in 1971 [4], discriminating audible types for these three basic damage modes using an AE system. After forty years, Godin et al. [5] conducted mapping of cross-ply glass/epoxy composites during tensile tests. They have classified four different acoustic signatures of failure and determined four conventional analyses of AE signals. 

Typical waveforms with A-Type (slow increase times at about 10–20 µs) signals associated with matrix cracking, B-Type (sharp rising, lasted for 10 µs and abruptly decreasing) with fibre/matrix interface de-bonding, C-Type associated with fibre failure, and D-Type (long rising times, high amplitudes, and very long durations) with delamination [5]. The most popular methods to identify damage are identification by signal amplitude distribution (signal strength) and by signal frequency. Table 1 and Table 2 show a comparison between the amplitude and the frequency distribution model that were encountered in the literature.

All of these studies show the difficulty of identifying damage modes for 2D composites and becomes more complicated for 3D woven composites. Only a small amount of investigation has been reported for monitoring evolution of damage and ultimate failure in 3D woven composites. Li et al. [15] studied AE signals for 3D non-crimp orthogonal woven glass/epoxy composites from cluster analysis point of view. These clusters are based on different parameters of peak amplitude, peak frequency, and RA value (rise time divided by peak amplitude). From their investigation, cluster 1 (low frequency, low amplitude events) and 2 (moderate frequency, low amplitude) is correlated to matrix cracking, cluster 3 (low to moderate frequency with high amplitude) with fibre and matrix de-bonding, and cluster 4 (high frequency) with delamination and fibre breakage. Lomov et al. [25] investigated AE response in 3D non-crimp orthogonal woven carbon/epoxy composites undergone damage.

However, identifying cracking in the matrix or fibre in addition to delamination need to be investigated further if AE is to be used as an inspection tool in SHM of 3D woven composites. Hence, the present study (qualitative and quantitative) of 3D angle-interlock woven composite damages using AE piezoelectric sensors is undertaken. As these structural woven fabrics are attracting the attention of the aerospace industry, the monitoring of initiation and progression of transverse matrix cracking is of considerable interest and importance, since they can lead to delamination and fibre breakage, which result to ultimate failure.

### 1.2. Guided Waves

Guided waves are very widespread in SHM applications: Guided waves are important for SHM applications because they have the ability to travel without much energy loss over large areas. This property makes them well suited for ultrasonic inspection of bridges, aircraft, ships, missiles, pressure vessels, pipelines, etc. In plates, ultrasonic guided waves propagate as Lamb waves and as shear horizontal waves (SH). Ultrasonic guided waves in plates were first described by Lamb (1917). A detailed study of Lamb waves has been given by Viktorov [26], Achenbach [27], Graff [28], Rose [29] and Dieulesaint and Royer [30]. Lamb waves are of two varieties, symmetric modes (S0, S1, S2...) and anti-symmetric modes (A0, A1, A2...). At low values of the frequency-thickness product, *fd*, the first symmetric mode, S0, resembles axial waves whereas the first anti-symmetric mode, A0, resembles flexural waves. The choice of Lamb waves is justified by their many advantages; they have the power to energize the entire thickness of the plate and offer the possibility of detecting internal defects at various depths. However, Lamb waves present some difficulties: they are dispersive, and also several modes can propagate at different speeds at a given frequency. Work has been done to establish analytically the dispersion curves in isotropic plates [30,31], to validate the results experimentally and to study the effect of dispersion over long distances [32]. Lamb wave propagation was used by many authors [33,34,35] using piezoelectric disks as transmitters and receivers to measure the changes in the signal received from a structure having a defect. However the signal processing is complex due to multiple reflections. Today the majority of work concerns the propagation of Lamb waves in thin isotropic structures. For this reason, it is very important to study the Lamb wave propagation from an acoustic emission point of view in 3D composite materials to understand the difficulties in analysing these waves in order to be able to qualify and quantify the defects in such structural configurations.

## 2. Materials Presentations and Experimental Set-Up

**Figure 1:**
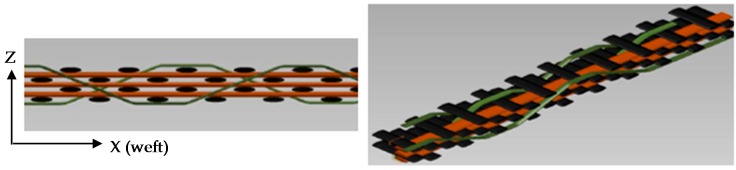
A schematic of 3D Angle Interlock Woven Composite (through thickness and planar view) (orange: weft; black: warp; green: binder yarn) (Binder yarn goes all the way through-the-thickness, z-axis, and then returns back).

In this study, a 3D angle interlock (AI) S2 glass woven composite plate with through-thickness binding was infused using bi-functional epoxy resin (LY564) and hardener (XB3486) supplied by Huntsman. In the AI configuration, the binder goes all the way through-the-thickness and then returns back. According to the binding pattern, shown in Figure 1 one binder yarn is inserted after every three layers of weft (yarn). This structure consists of 4 layers of warp (fibres parallel to weaving direction or at 0°) and 3 layers of weft (fibres transverse to weaving direction or at 90°), which are held together by the binders (through-thickness fibres) inserted in the weft direction at regular intervals as illustrated in Figure 1.

**Figure 2:**
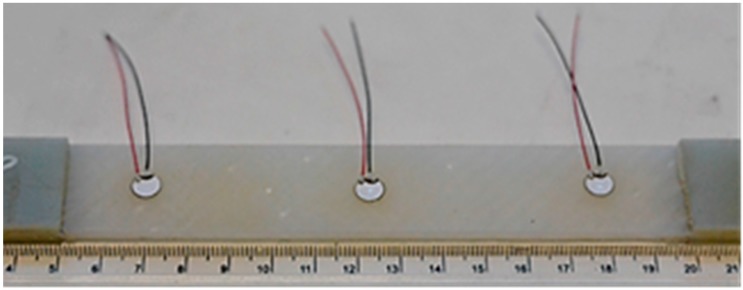
PWAS bonded on a 3D angle interlock glass fibre tensile specimen for acoustic emission.

Tensile testing was carried out according to ASTM standard D3039 [36], on specimens 250 mm long (with a gauge length of 50 mm) and 25 mm wide. The tensile load was applied in the weft direction. A non-contact video extensometer was used to measure the strain developed while the specimen was loaded in an Instron 5982 R2680 testing machine (Norwood, MA, USA). Three piezoelectric wafer active sensors (PWAS) bonded on the specimen were acting as AE receivers, Figure 2.

To develop only transverse cracks, the specimen was loaded up to 20% of its ultimate strength (σ_f_). During loading, acoustic emission signals were recorded and the PWAS were able to pick up AE signal of good strength at a frequency range 100–700 kHz. The acquisition of the signals was performed using software ‘AEWin’ from Mistras with a sampling rate of 10 MHz and 20 dB pre-amplification. The AE PWAS sensors used in this study were provided by Steminc, further details in [37].

## 3. Angle Interlock Cracking Simulation

Fibre reinforced composite materials exhibit mostly a linear elastic behaviour similar to brittle materials up to the final failure specially when loaded along the fibre direction in tension. This mainly occurs because the most significant contribution for the load carrying capacity of these materials depends on the longitudinal fiber properties and strength. Even if some progressive failure occurs in the matrix or transverse cracking, still composites can carry the load up to the fiber failure along the loading direction. From this perspective, linear elastic fracture mechanics can be employed to describe and analyse the fracture “cracking” of fiber reinforced composites [38]. Any finite fracture that occurs in a composite material is governed by the first law of thermodynamics. The energy dissipated due to crack formation normalized by the surface area of the newly formed crack is known as the energy release rate (G). Transverse cracking and local delamination are two common types of cracking mechanisms that occur in composite materials. In order for any of these matrix cracking mechanisms to exist [38], the strain energy release rate associated with each damage mechanism (G) should exceed its critical strain energy density “toughness” $\left(G_{c}\right)$. Therefore, the question always is how to determine the energy release rate (G) for heterogeneous materials like composites. The strain energy release rate for composite materials is calculated as [39]:

(1) $$G=-\frac{\Delta E}{\Delta A}$$

where ΔE is the strain energy released due to the cracking formation. This is determined by subtracting the strain energy density of a cracked cell from the strain energy density of non-cracked cell while ΔA represents the area of the cracked surface. Strain energy release rate actually defines the potential locations for crack formation along the yarn or its cross section. Cracks are more likely to form in locations where the strain energy release rate is high.

For composite materials, the strain energy density can be calculated as function of the applied strain/ stress. Therefore, the strain energy density components can be calculated as follows [40]:

(2) $$e_{ij}=\frac{1}{2V}\int_{v}\frac{\sigma_{ij}^{2}}{E_{ij}}$$

where V is the volume of the (ply/yarn/laminate) determined as the cross-sectional area multiplied by the thickness, $\sigma_{ij}$ is the ij component of stress and $E_{ij}$ is the corresponding Young’s modulus (𝑖 = 𝑗) or Shear modulus (𝑖 ≠ 𝑗).

**Figure 3:**
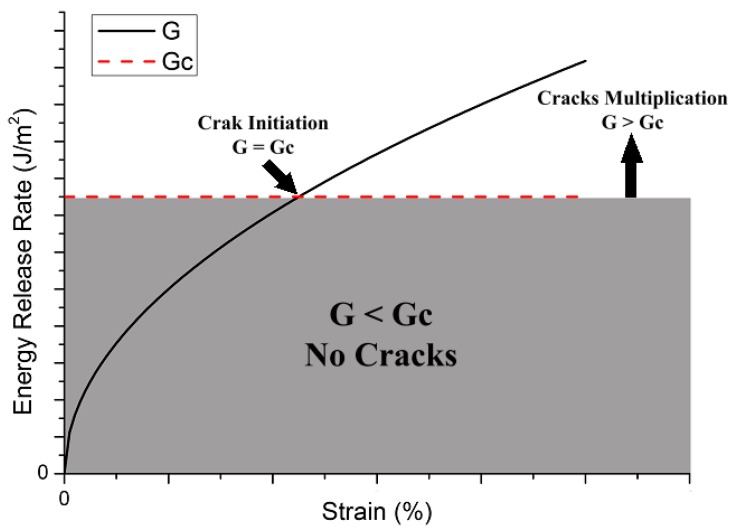
Graphical representation of the finite fracture mechanics theory.

Figure 3 is a graph to illustrate the theory behind the finite fracture mechanics. The toughness of the material for a specific cracking mechanism $\left(G_{c}\right)$ is a material property which is constant while the energy release rate increases with increasing applied stress/strain. Once the energy release rate associated with a specific cracking mechanism exceeds the critical value, crack formation and damage evolution starts.

On more issue regarding the fracture of composite materials is that the fracture occurs due to multiplication of cracking events rather than growth of a single crack. Therefore, the fracture response of composite materials is more like discrete instantaneous crack propagation. For further details about the application of finite fracture mechanics of composite materials, the reader is referred to [38].

The 3D Angle Interlock Woven Composite (3DAWC) (Figure 1) is modelled as a (0/90) cross-ply laminate since the crimp mostly occurs at the interlacement points between the weft and binder yarns [40]. In order to check the effect of this simplification on the in-plane properties of the 3DAWC, analytical homogenization technique “orientation averaging model” is used to calculate approximately the elastic material properties [41,42] and compare it with the measured data obtained. As shown in Table 3, good agreement between the experimental and analytical model is obtained while the last column represents the difference between the calculated values with and without the binder yarns, confirming that the z-yarns have negligible effect on axial stiffness. This result justifies the representation of the 3D woven architecture by a cross-ply (0/90) laminate used in the AE simulation, see Section 4.

A larger impact of the through-the-thickness reinforcement is expected on the interlaminar fracture toughness rather than in-plane stiffness properties. An almost 14% increase in E33 modulus is predicted when the binder yarns are considered in the analysis.

**Figure 4:**
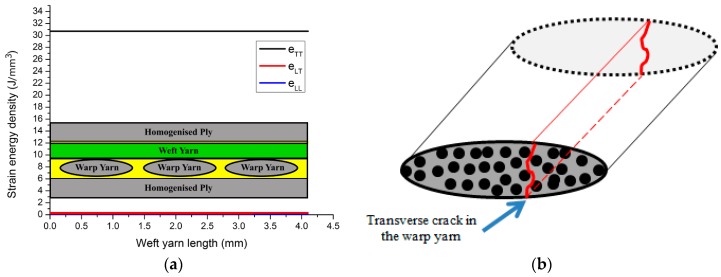
(**a**) Strain energy release rate along weft yarn (TT: Transverse component; LT: shear component; LL: axial component); (**b**) crack on a warp yarn cross section (Transverse crack).

To determine which constituent part of the 3D woven will experience cracking in the case of uniaxial tension, strain energy density components are calculated for the 3D AI woven composites unit cell when applying 1% strain along the weft direction. The finite element model is run using the COMSOL Multi-physics software package. Figure 4 shows that the transverse component $e_{TT}$ of the strain energy density is the highest when compared to the longitudinal $e_{LL}$ and shear $e_{LT}$ components. This implies that the strain energy release rate for the transverse component is the one that leads to matrix cracking in the weft yarn under this loading condition. In addition, having a constant energy release rate along the whole yarn length, it suggests that there is no preferable location within the yarn for the crack to start from. This also means that once a crack is initiated in the yarn, it grows instantaneously through the thickness and along the whole yarn length. The complete study of damage mechanisms is well explained and characterised in references [43,44]. 

Matrix cracking is a phenomenon that generates a motion which is essentially in plane. The motion of the crack faces is parallel to the plane of the specimen. It can thus be expected that matrix cracks will generate AE waves which contain a predominant extensional mode. Fibre fracture follows the same general behaviour and should therefore also be characterised by a large extensional mode [45].

A delamination is a damage phenomenon that generates a motion, which is essentially out of plane. In this case, the motion is perpendicular to the plane of the plate. Delaminations should thus generate AE waves, which contain a dominant flexural mode. Fibre/matrix debonding follows the same behaviour and should also be characterised by a large flexural mode. It should be noted that delamination and fibre/matrix debonding can be also driven by shear stresses where there is no crack opening but crack sliding making it more difficult to detect non-destructively.

## 4. Acoustic Emission Simulation

**Figure 5:**
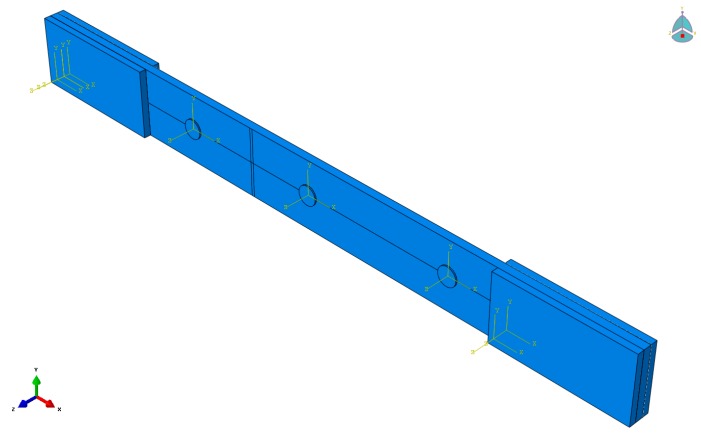
ABAQUS model of the homogenised 3D woven composite with 3 PWAS bonded on the top to record the AE events from the surface simulated transverse crack.

Simulation of AE was realised using the ABAQUS/implicit software (Dassault Systèmes, Vélizy-Villacoublay, France), which has multi-physics piezoelectric elements. FEM modelling was used to simulate the elastic wave emitted by the transverse crack growth. These can be used to compare with the results obtained from the experiment. The ABAQUS model is shown in Figure 5. This structure, consisting of 4 layers of warp (at 0°), 3 layers of weft (or at 90°), and held together by the binders (through-thickness fibres) are homogenised. Two elements per ply are used. Eight nodes linear piezoelectric brick element were used to simulate the PWAS. Implicit solver methods of solution are used in order to simulate the real voltage/amplitude received signal [46]. The use of multi-physics finite element method (MP-FEM) is explored to model the reception of the elastic wave as electric signal recorded at a PWAS receiver (R-PWAS).

The piezoelectric material properties were assigned to the PWAS as described in reference [37]:

(3) $$[C]=\left(\begin{array}{cccccc} 97 & 49 & 49 & 0 & 0 & 0\\ 49 & 97 & 44 & 0 & 0 & 0\\ 49 & 49 & 84 & 0 & 0 & 0\\ 0 & 0 & 0 & 24 & 0 & 0\\ 0 & 0 & 0 & 0 & 22 & 0\\ 0 & 0 & 0 & 0 & 0 & 22 \end{array}\right)\left(\text{GPa}\right)$$

(4) $$[\varepsilon ]=\left(\begin{array}{ccc} 947 & 0 & 0\\ 0 & 947 & 0\\ 0 & 0 & 605 \end{array}\right)\times 10^{-8}\left(F/m\right)$$

(5) $$[e]=\left(\begin{array}{cccccc} 0 & 0 & 0 & 0 & 12.84 & 0\\ 0 & 0 & 0 & 12.84 & 0 & 0\\ -8.02 & -8.02 & 18.31 & 0 & 0 & 0 \end{array}\right)\left({C/m}^{2}\right)$$

where [C] is the stiffness matrix; [ε] is the dielectric matrix and [e] is the piezoelectric matrix. PWAS has a density of ρ = 7600 Kg/m^3^, diameter of 7 mm, and thickness of 500 µm. The 3D composite properties are shown in Table 3 and the Rayleigh damping coefficients from reference [2] are used. It should be noted that these Rayleigh damping coefficients may have an effect on the wave amplitude of the signal but not the shape of the waveform, which is used in characterizing the damage mode.

**Figure 6:**
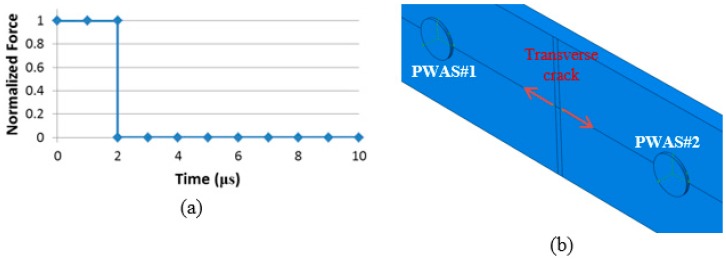
(**a**) Source function used: at time zero the force step up from 0 to a nominal value 1, and then return to 0 at 2 μs; (**b**) two-point source force to simulate the energy release by the transverse crack.

The maximum frequency of interest was chosen at around 600 KHz. For 600 KHz, a time interval of 0.1 µs and an element size about 0.5 mm in the composite plate are required to achieve an error on wave velocity below 5% [46,47]. A step excitation was used as shown in Figure 6a. To simulate the energy released by the transverse crack a two-point source force was applied between PWAS#1 and PWAS#2 at the surface of the specimen as illustrated in Figure 6b. A shear force, parallel to the crack could also be used, but would have no effect on the shape of the signals received by the PWAS. The end of the specimen is fixed to represent the real boundary conditions of the tensile test. However, the tensile load is not simulated.

## 5. Results and Discussion

### 5.1. Multi-Physics Finite Element Simulation

**Figure 7:**
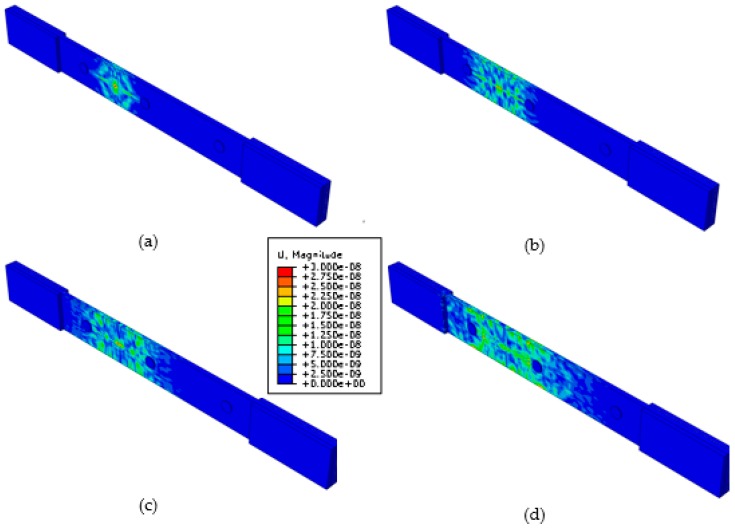
Snapshot of the MP-FEM simulation of guided waves generate by a pair of point forces simulating an acoustic emission by the transverse crack in a 3D angle interlock composite tensile specimen at (**a**) 10 μs; (**b**) 20 μs; (**c**) 30 μs; (**d**) 40 μs.

Figure 7 shows image snapshots of overall displacement amplitude of the guided wave pattern in the plate taken at 10-μs intervals. Multiple guided waves modes are present. At *t* = 10 μs, one sees the waves just starting from the transverse crack. By *t* = 20 μs, most of the wave has already being reflected from the edges of the tensile specimen which will complicated the analysis of the received signal due to Lamb waves mode conversion.

**Figure 8:**
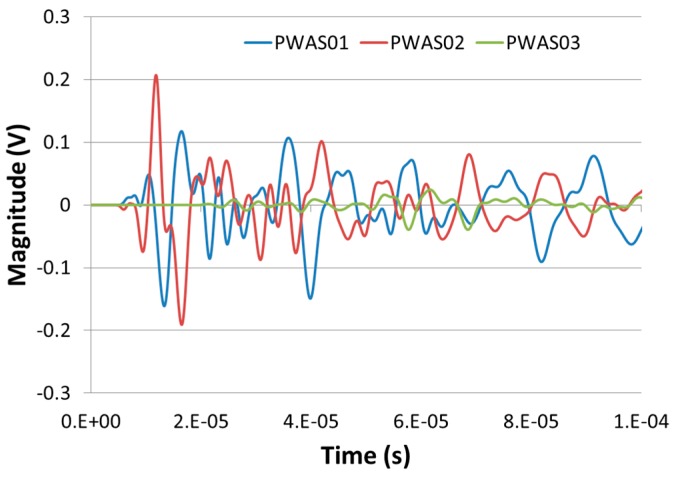
Simulated signal received: Output voltage against time for PWAS#01, 02, and 03.

The simulated AE signal caused by the simulated transverse crack excitation as captured at PWAS#1, 2, and 3 is shown in Figure 8. The magnitude of the received signal from PWAS#3 (in green) decreased dramatically due the damping effect introduced in the model. 

To better understand these signals, the discrete wavelet transform (DWT) is used. The DWT of a time signal s(t) is the result of the convolution product between the signal s(t) and a family of “daughter wavelets” $\gamma_{m.k}(t)$,

(6) $$DWT_{m,k}=\int_{0}^{\infty }s(t)\gamma_{m,k}(t)dt$$

**Figure 9:**
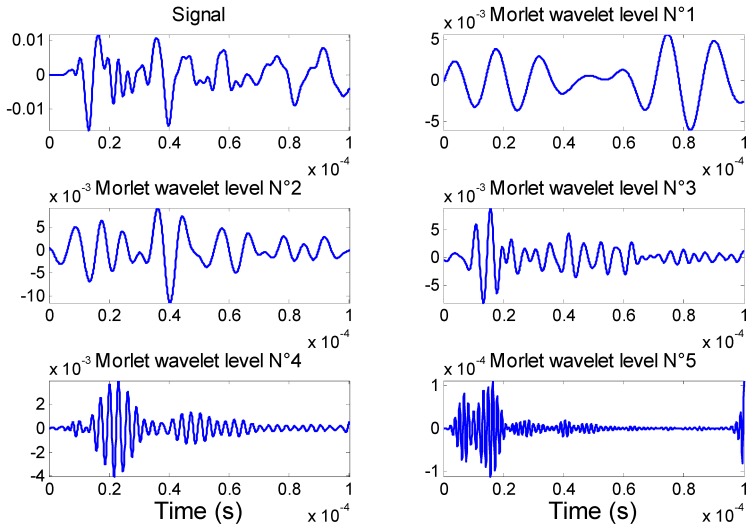
Discrete wavelet transform of the simulated signal received by the PWAS#1.

The main particularity of the DWT is that the result obtained with each daughter wavelet corresponds to the time behaviour of the signal in a frequency band corresponding to dilatation factor *m*. Each response is called the decomposition level. A number of different bases have been proposed to construct a family of wavelets. A good solution for analysis and decomposition can be obtained with the Morlet wavelet. The application of discrete wavelet analysis to the acquired AE signals resulted in its decomposition into six different levels. Each level represents a specific frequency range, and the frequency range increases with increasing wavelet level. The decomposed AE signals in level 1 to 5 are shown in Figure 9 for the PWAS#01.

**Figure 10:**
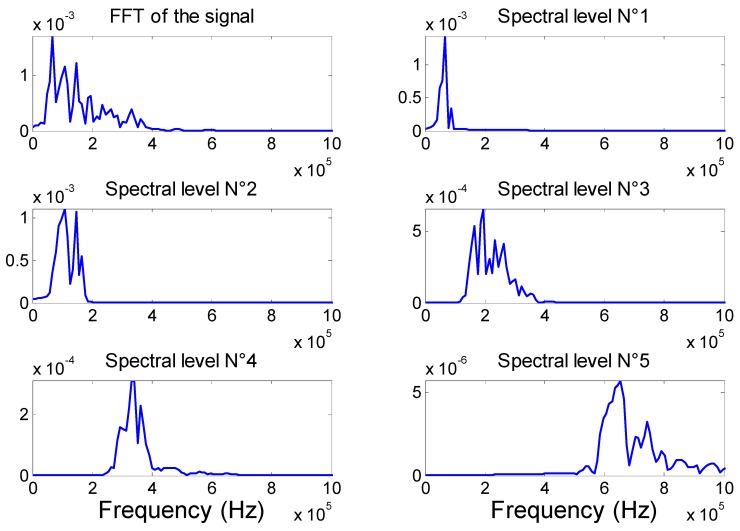
Frequency spectra for the different wavelet level (PWAS#1).

The Fourier spectrum of the Figure 9 signals is shown in Figure 10. The frequency spectra for DWT levels 1 through 5 are centered at about 68 kHz, 120 kHz, 200 kHz, 340 kHz, and 650 kHz, respectively. At frequencies 68 kHz, 120 kHz, and 200 kHz (Morlet wavelet levels 1 and 2), three modes exist, the fundamental symmetric mode (S0), the fundamental anti-symmetric mode (A0), and the fundamental shear mode (SH0). However, with the PWAS receiver geometry and properties, the SH mode cannot be caught by these sensors [2]. Moreover, based on the tuning study, at 68 kHz the amplitude of the A0 mode is much higher than the S0 mode, and its travel speed is slower. At 120 kHz, the amplitude of A0 and S0 are almost the same, and at 200 kHz, the amplitude of the S0 is higher than the A0. To conclude, the component at low frequency (below 140 kHz) is dominated by the fundamental anti-symmetric mode A0. At 340 kHz (Morlet wavelet level 3), four modes are existent, S0, A0, A1 and S1; at 650 kHz (Morlet wavelet level 4), six modes are present, S0, S1, S2, A0, A1, and A2. Therefore, at these frequencies, the distinction of the different wave packets and the signal processing are very complex. Moreover, the amplitude is distributed such that it is the highest in level 1 and lowest in level 5 as shown in Figure 9. The FFT of the original signal shows that the amplitude of the signal is higher for the frequency lower than 160 kHz, which mean that the transverse crack develops more flexural (i.e., A0) than extensional (i.e., S0) motion.

However, Surgeon and Wevers [42] mentioned that matrix cracks will generate AE waves which contain a predominant extensional mode (i.e., S0 mode). It might be explained by the symmetry of the transverse crack, which is maybe not the case in our experiments.

**Figure 11:**
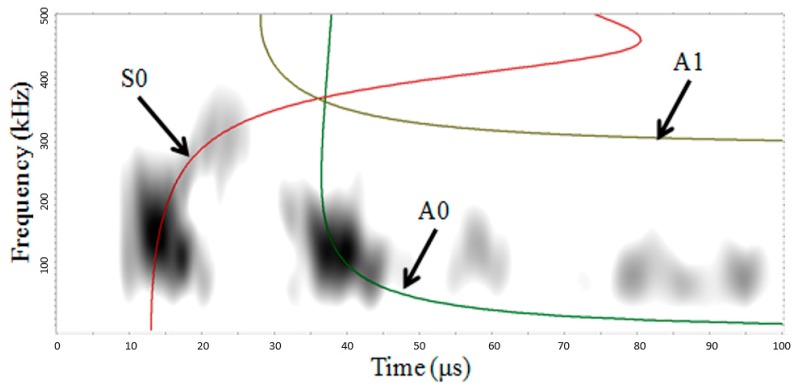
Superimposed symmetric mode and anti-symmetric modes after converting group velocity to time based on the propagation distance. Light and dark grey correspond to simulated AE activity.

Figure 11 shows the continuous wavelet transform (CWT) magnitude as a function of frequency versus time. The CWT were calculated with AGU-Vallen Wavelet, a freeware software program [48]. This program has a Gabor function as the “mother” wavelet. Figure 11 shows the analytical dispersion curves with the three lowest modes (S0, A0, and A1) superimposed on the CWT plot. The colour scale is a linear scale with black representating the highest magnitude and white the lowest or zero-magnitude region. Clearly, Figure 11 shows the presence of AE signal energy in portions of mainly two modes, A0 and S0. The CWT shows how the signal energy is distributed as a function of frequency, time (or group velocity), and mode. Figure 11 shows that the simulated AE source has the greatest concentration (most black color) of energy is the fundamental anti-symmetric mode A0 in a frequency range of 50 to 250 kHz. Another large amplitude region of the CWT is the part of the fundamental symetric mode S0 in a frequency range 50 to 300 kHz. This demonstrates that the AE signal energy is not uniformly distributed between the modes; it is also not uniformly distributed as a function of frequency along each of the dominant modes.

The above discussion proves that the waveforms features (duration time, amplitude, time-frequency spectrum) are useful to illustrate the characteristics of AE signal and distinguish the different AE signals associated with various possible failure modes in the specimens. Moreover, PWAS#2 and PWAS#3 obtained similar trend to the PWAS#1.

### 5.2. Experiments

**Figure 12:**
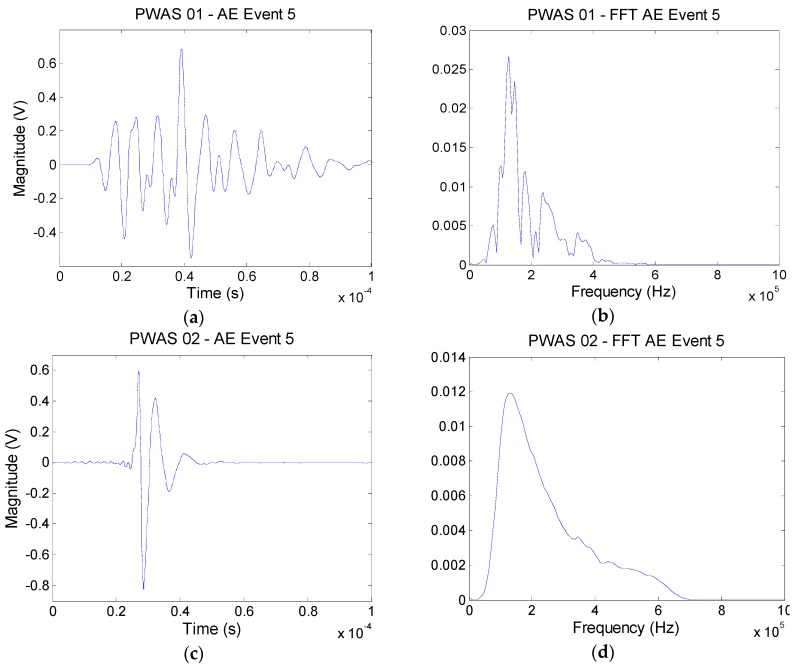

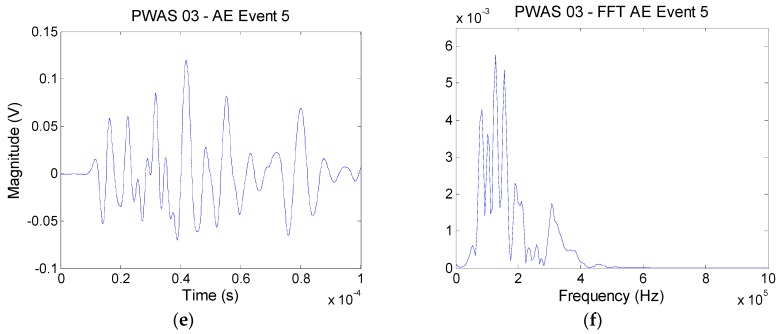
Typical experimental AE waveforms and Fourier Transform from a transverse crack in 3D AI recorded from (**a**,**b**) PWAS#1; (**c**,**d**) PWAS#2; (**e**,**f**) PWAS#3.

As mentioned in Section 3, at this applied tensile load only transverse cracking occurs in the studied specimen. Figure 12 shows typical AE waveforms received by the PWAS#1, #2, and #3, and the associated Fourier transform. 

In this particular example, the transverse crack occurs closer to PWAS#2 than the other sensors. This signal looks sharper and stronger than those obtained by PWAS#1 and #3. Masmoudi et al. [12] classified these very energetic signals with amplitude above 94 dB to fibre breaking. However, in theory, no fibre breakage should occur, only transverse crack in the warp yarn should develop as previously simulated. In the next section, the stress amplification factor (SAF) is introduced to explain this typical fibre breakage waveform. The amplitudes of this particular event are 96, 98, 81 dB for PWAS#1, #2, and #3, respectively. The amplitude decreases with the travel length due to the high damping coefficient in this 3D AI composite materials. 

**Figure 13:**
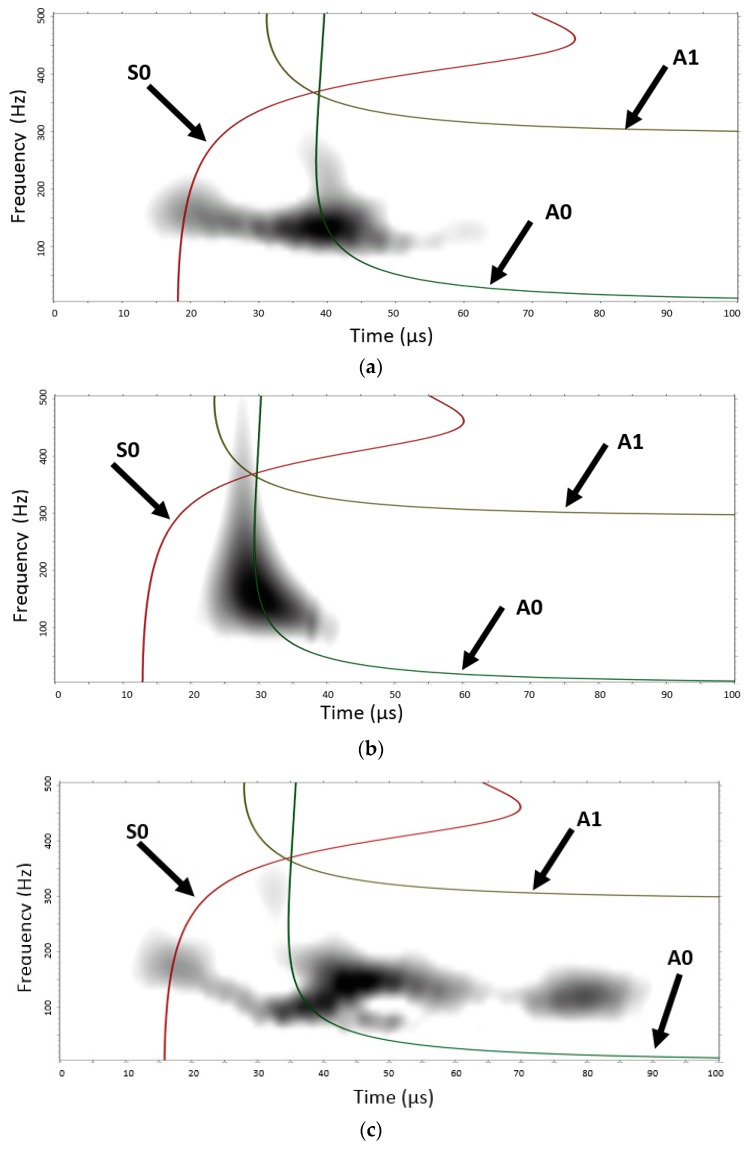
Superimposed symmetric mode and anti-symmetric modes after converting group velocity to time based on the propagation distance for the experimental received signal: (**a**) PWAS#1; (**b**) PWAS#2; (**c**) PWAS#3.

Figure 13 shows the CWT magnitude as a function of frequency versus time and shows the anlaytical dispersion curve with the three lowest modes (S0, A0, and A1) superimposed on the CWT plot of the typical AE waveforms recorded from PWAS#1, #2 and #3. The colour scale is a linear scale with black representating the highest magnitude and white the lowest or zero-magnitude region. The CWT shows how the signal energy is distributed as a function of frequency, time (or group velocity), and mode. Figure 13a shows the presence of AE signal energy in portions of mainly two modes, A0 and S0 for the PWAS#1 which is in agreement with our MP-FEM results shown in Figure 11. The experimental AE source has the greatest concentration of energy is the fundamental flexural mode A0 in a frequency range of 80 to 300 kHz (the simulated AE event is in a frequency range of 50 to 200 kHz for the A0 mode). Another large amplitude region of the CWT is the part of the fundamental extensional mode S0 in a frequency range 110 to 220 kHz (the simulated AE event is in a frequency range of 50 to 300 kHz for the S0 mode). Figure 13b shows the presence of AE signal energy in portions of only one mode, A0 for the PWAS#2. This experimental AE source is the fundamental flexural mode A0 in a frequency range of 80 to 500 kHz with a higher concentration between 120 to 250 kHz. During this typical event, damage occurs close to PWAS#2 and so the wave does not have time to travel over long distance. Moreover, this waveform is assimilited to a micro-fibril breakage (binder yarn) with very high energy which shadow all the reflection waves from the edge. Figure 13c shows the presence of experimental AE signal energy in portions of mainly two modes, A0 and S0 for the PWAS#3. 

Figure 13c shows that the AE source has the greatest concentration of energy is the fundamental flexural mode A0 in a frequency range of 60 to 230 kHz (the simulated AE event is in a frequency range of 50 to 200 kHz for the A0 mode). Another large amplitude region of the CWT is the part of the fundamental extensional mode S0 in a frequency range 130 to 250 kHz (the simulated AE event is in a frequency range of 50 to 300 kHz for the S0 mode). Because the experimental AE event occur far away from the PWAS#3 several reflections are also visible. This demonstrates that the AE signal energy is not uniformly distributed between the modes; it is also not uniformly distributed as a function of frequency along each of the dominant modes.

In summary, it seems that transverse crack (simulated and experimental) generates a predominant flexural mode A0 and also a less energetic extensional mode S0. Moreover, the micro-fibril breakage (in the binder yarn) at the tip of the transverse crack (typical waveform—Figure 12c) generates only the fundamental flexural mode A0. This conclusion is in disagreement with previous study [45]. It might be explained by the non-symmetry of the damage which is maybe not the case in the others experiments.

Moreover, the frequency of these signals show clearly two major components, the first one between 70 and 180 kHz and the second one between 200 and 400 kHz for PWAS#1 and #3. 

**Figure 14:**
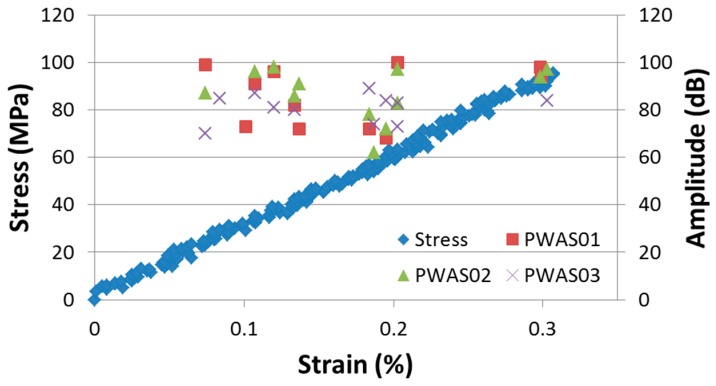
Applied stress-strain curve and the PWAS amplitude for each AE events (transverse cracks and micro-fibril breakage). Ultimate failure strain = 1.3%.

The high frequency and the low frequency component correspond to the wave’s extensional mode S0 and to the flexural mode A0, respectively, as showed in the MP-FEM simulation. This flexural mode A0 has higher amplitude than the extensional S0 mode. It seems that the transverse cracks generate more flexural motion than extensional motion. This presence of a flexural mode would indicate that the crack does not develop symmetrically about the mid-plane of the 3D AI laminate. The crack initiation for the loading in weft direction occurs in the range of applied strain 0.07%…0.1% (Figure 14, showing the data for weft direction of loading), a relatively low level of strain. The amplitude for each AE event (i.e. transverse crack) is between 60 and 100 dB. The signals with lower amplitude were assimilated into noise.

These experimental and simulated results have proven that transverse matrix cracking signals do exhibit a clear fundamental flexural A0 mode. In most cases, however, the extensional mode was also clearly present. For the transverse matrix crack signals this is caused by their asymmetric growth through the thickness. Matrix cracks most often initiate at one of the outer plies and grow through the thickness to the other side of the specimen. These results in a particle motion which is in plane, but asymmetric about the mid-plane, thus resulting in a flexural mode. The large flexural mode observed during this test can be explained by the same principle: transverse cracks will occur preferably in the zone of maximum tensile stress. AE waves generated there will thus cause an in plane motion, but the motion will be asymmetric about the mid-plane. This will again result in a flexural component. 

### 5.3. Stress Amplification Factor

**Figure 15:**
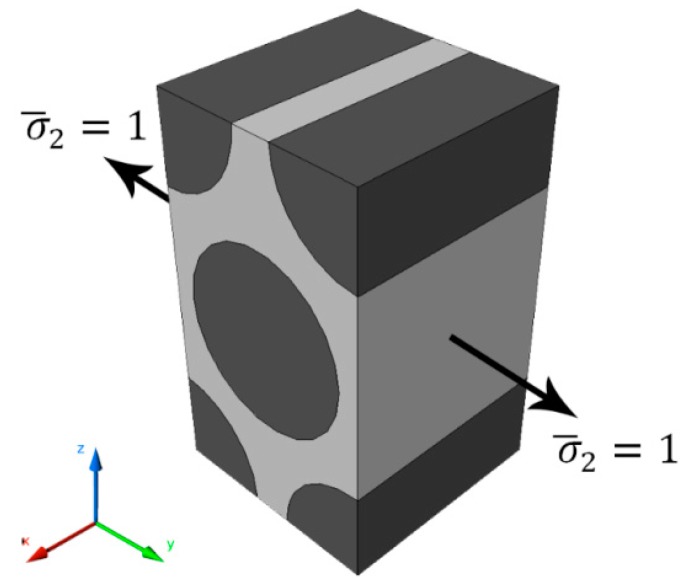
Fibre hexagonal array unit cell subjected to unit load.

On the micro-mechanical analysis, the external applied stress and the local stress within the material is not the same due to the difference in the material properties of the material constituents. A random fibre distribution in a yarn can be simplified by a unit cell of a hexagonal array distribution. When this unit cell is subjected to an external load as shown in Figure 15, the fibre and matrix will experience different stresses resulting in a stress concentration within the unit cell. Therefore, it is obvious that if an external uniform unit load is applied on the boundary, the stresses within the unit cell are not unity. 

Cesar et al. [49] reports in that there are amplification factors that relate the macroscopic $\left(\bar{\sigma }\right)$ uniformly distributed unit load to the micromechanical stresses (σ) within the unit cell:

(7) $$\sigma =M_{\sigma }\bar{\sigma }+A_{\sigma }\Delta T$$

$M_{\sigma }$ and $A_{\sigma }$ are two matrices that contain the mechanical and thermal amplification factors, respectively while ΔT represents the change in room temperature. The $M_{\sigma }$ matrix can be calculated by applying unidirectional unit load each at a time. Therefore, for instance the first step is applying ${\bar{\sigma }}_{1}=1$ to get the first column of the matrix and so on. The stress amplification factor $M_{\sigma }$ within the unit cell will vary at each point so it will end up having a contour map of the stress amplification factors over the representative volume element (RVE size: 10 mm × 5 mm). The same technique can be applied to obtain the strain amplification factors $M_{\varepsilon }$ and $A_{\varepsilon }$:

(8) $$\varepsilon =M_{\varepsilon }\bar{\varepsilon }+A_{\varepsilon }\Delta T$$

**Figure 16:**
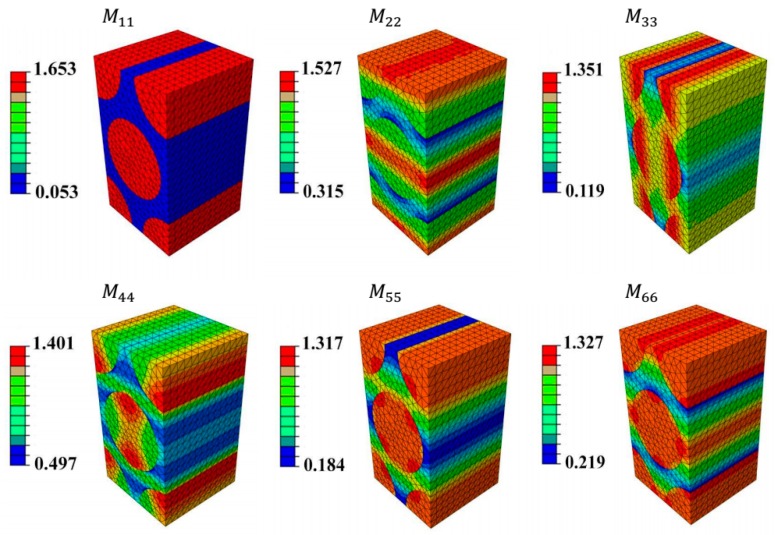
Diagonal elements of SAF tensor for hexagonal unit cell.

Further details regarding applying the boundary conditions and calculating the SAF can be found in [49,50]. After obtaining the stress amplification factors, a full description of the microscopic stress distribution within the unit cell can be determined as shown in Figure 16.

Just for clarification, only the diagonal elements of the stress amplification factor tensor $\left(M_{\sigma }\right)$ are listed below. It is clear that the maximum stress is approximately 1.6 when the external applied load on the boundary is unity. The same concept has been observed experimentally, on the meso-scale, for 3D woven composites loaded in tension using image correlation [41]. This could justify why micro-fibril breakage is detected by AE event even when the applied global stress/strain is way below the ultimate strength or failure strain of fibres on the microscale or on the mesoscale. In case of a coupon specimen tested in tension, this applies for the loading direction $\left(M_{11}\right)$ and both transverse directions $\left(M_{22}\&M_{33}\right)$ due to the Poisson’s contraction effect; further work is required to capture more accurately the effect of the 3D fibre architecture on damage evolution.

## 6. Concluding Remarks

Transverse cracking in the warp yarn was detected and quantified in a 3D angle interlock woven glass composite plate during a tensile test using piezoelectric wafer active sensors bonded on the surface of the sample. The angle interlock cracking simulation have shown that the transverse component of the strain energy density is the highest when compared to the longitudinal and shear components. This implies that the strain energy release rate for the transverse component is the one that leads to transverse matrix cracking in the weft yarn under tensile loading. AE simulation has been conducted with the MP-FEM approach. The AE event was simulated as a pulse of defined duration and amplitude. The simulated electrical signal was measured at a receiver PWAS using the MP-FEM capability with the piezoelectric element. Morlet wavelet transforms and their FFT frequencies were used to process the signal in order to define and separate the different modes that composed the AE signal. These results show that the amplitude of the AE signal depends on the distance between the crack and the sensor (affected by damping). Moreover, simulated and experimental transverse cracking generates a predominant fundamental flexural mode A0 and also a less energetic fundamental extensional mode S0. Moreover, the binder yarns at the tips of the transverse crack might break which is represented by a typical AE waveform (shape and energy). This micro-fibril breakage generates only the fundamental flexural mode A0. In addition, the stress amplification factor was developed to justify why transverse matrix cracking and micro-fibril breakage is detected by AE event even when the applied global stress/strain is way below the ultimate strength or failure strain of matrix/fibres on the microscale or on the mesoscale. 

In the near future, more work needs to be done on (a) calibrating the MP-FEM modelling of guided wave for accurate representation of physical phenomenon; (b) simulate the real energy release of crack growth using XFEM or VCCT model; (c) better understand the multi-modal guided wave propagation in complex 3D woven composite plates and identify more effective wave-tuning methods and signal processing algorithm for damage identification and localisation. A complete study on the guided wave propagation and the attenuation effect is also required in order to increase the accuracy of the results. 

Although some good progress has been demonstrated, there are still some outstanding questions that need to be answered. A complete experimental research program and a MP-FEM method need to be fully performed in order to better understand the damage evolution (that includes multiple matrix cracks, delamination, and fibre breakage) and ultimate failure of these 3D AI glass composite plates.

## Figures and Tables

**Table 1:** Amplitude distribution according to the damage mechanism in composite materials.

Ref.	Matrix Cracking	Interface Decohesion (Fibre/Matrix)	Fibre/Matrix Friction and Fibres Pull-Out	Fibres Breakage
[6]	30–45 dB	45–55 dB	–	>55 dB
[7]	60–80 dB	70–90 dB	–	–
[8]	50 dB	–	–	–
[9]	40–70 dB	–	–	60–100 dB
[10]	40–55 dB	–	>80 dB	–
[11]	33–45 dB	50–68 dB	69–86 dB	87–100 dB
[12]	40–78 dB	72–100 dB	–	95–100 dB
[13]	40–55 dB	60–65 dB	65–85 dB	85–95 dB
[5]	35–80 dB	50–80 dB	70–100 dB	–
[14]	<70 dB	<60 dB	–	–
[15]	35–55 dB	55–100 dB	–	35–80 dB
[16]	40–60 dB	50–70 dB	80–100 dB	80–100 dB

**Table 2:** Frequency distribution according to the damage mechanisms in composite materials.

Ref.	Matrix Cracking	Interface Decohesion (Fibre/Matrix)	Fibre/Matrix Friction and Fibres Pull-Out	Fibres Breakage
[17]	50–150 kHz	–	–	140–180 kHz
[18]	30–150 kHz	30–100 kHz	180–290 kHz	300–400 kHz
[19]	80–130 kHz	–	250–410 kHz	250–410 kHz
[14]	~300 kHz	–	300 kHz	>500 kHz
[20]	50–180 kHz	220–300 kHz	180–220 kHz	>300 kHz
[21]	90–110 kHz	–	200–300 kHz	>420 kHz
[22]	<50 kHz	200–300 kHz	500–600 kHz	400–500 kHz
[23]	~140 kHz	~300 kHz	–	~405 kHz
[24]	200–600 kHz	200–350 kHz	0.7–1.1 MHz	>1.5 MHz
[15]	50–80 kHz	50–150 kHz	–	150–500 kHz

**Table 3:** Elastic material properties of 3D AI woven composites.

Mechanical Data	Experiment	With Binder	Without Binder	Difference (%)
$E_{1}$	18.52±0.87	17.85	17.33	2.91
$E_{2}$	24.83±1.51	24.00	23.48	2.16
$E_{3}$	–	12.74	11.00	13.65
$G_{12}$	–	5.18	4.95	4.50
$\nu_{12}$	–	0.31	0.32	0.68
*V_F_* (%)	$V_{F}=50.35\pm 0.26;\;V_{F}(\text{warp})=31.21\pm 0.26;\;V_{F}(\text{weft})=15.38\pm 0.36;\;V_{F}(\text{binder})=3.05\pm 0.33$			
